# Microalbuminuria After Kidney Transplantation Predicts Cardiovascular Morbidity

**DOI:** 10.3389/fmed.2021.635847

**Published:** 2021-04-12

**Authors:** Dana Bielopolski, Ruth Rahamimov, Boris Zingerman, Avry Chagnac, Limor Azulay-Gitter, Benaya Rozen Zvi

**Affiliations:** ^1^Department of Nephrology and Hypertension, Rabin Medical Center, Petah-Tikva, Israel; ^2^Sackler School of Medicine, Tel-Aviv University, Tel-Aviv, Israel

**Keywords:** kidney transplantation, albuminuria, urine collection, cardiovascular morbidity, proteinuria

## Abstract

**Background:** Microalbuminuria is a well-characterized marker of kidney malfunction, both in diabetic and non-diabetic populations, and is used as a prognostic marker for cardiovascular morbidity and mortality. A few studies implied that it has the same value in kidney transplanted patients, but the information relies on spot or dipstick urine protein evaluations, rather than the gold standard of timed urine collection.

**Methods:** We revisited a cohort of 286 kidney transplanted patients, several years after completing a meticulously timed urine collection and assessed the prevalence of major cardiovascular adverse events (MACE) in relation to albuminuria.

**Results:** During a median follow up of 8.3 years (IQR 6.4–9.1) 144 outcome events occurred in 101 patients. By Kaplan-Meier analysis microalbuminuria was associated with increased rate of CV outcome or death (*p* = 0.03), and this was still significant after stratification according to propensity score quartiles (*p* = 0.048). Time dependent Cox proportional hazard analysis showed independent association between microalbuminuria and CV outcomes 2 years following microalbuminuria detection (HR 1.83, 95% CI 1.07–2.96).

**Conclusions:** Two years after documenting microalbuminuria in kidney transplanted patients, their CVD risk was increased. There is need for primary prevention strategies in this population and future studies should address the topic.

## Introduction

Increased urine protein excretion is a validated risk factor for end stage kidney disease (ESKD), and cardiovascular morbidity and mortality (1–3). microalbuminuria defined as urine excretion between 30 and 300 mg albumin per 24 h, has been used as an early marker for diabetic kidney disease (4, 5), and has been recognized as an important marker for cardiovascular disease (CVD) in the diabetic and the non-diabetic populations (6, 7).

Following kidney transplantation recipients become a unique chimera, in which the renal vasculature is different from that of the other organs. This is an interesting setting to explore the link between microalbuminuria and CVD, as the kidney endothelium, that facilitate most of the leakage of protein into the urine, represents the donor rather than the recipient. A paper published in 2007, was the first to describe in kidney transplanted patients the correlation between albuminuria, mortality and eventually graft loss, stating that its prognostic strength surpasses that of renal function (8). The largest retrospective analysis published so far, implied that transplanted patients could manifest the same relationship between albuminuria and CVD. Weiner et al. showed that microalbuminuria, as evaluated by albumin creatinine ratio (ACR), had a non-significant trend for association with increased risk of CVD in patients after kidney transplantation (9). An analysis from our institute showed proteinuria, using urine dipstick, was significantly associated with an increased risk of major cardiovascular adverse events (MACE) (10). However, this association was not evaluated by the gold standard of timed urine collection. Our study sought to characterize the association between microalbuminuria, as evaluated by precise urine collection, and CVD risk in stable patients after kidney transplantation.

## Methods

**Figure 1:**
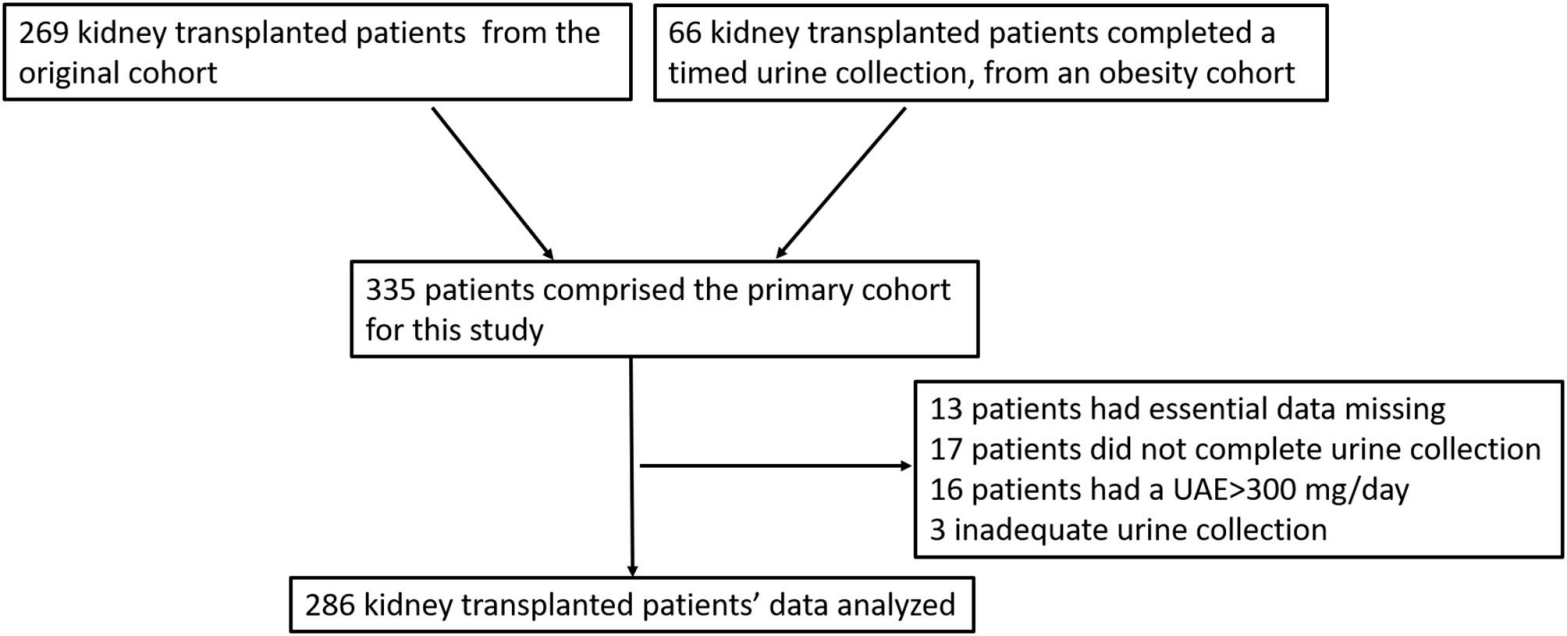
Consort diagram describing addition and reduction of patient to create the analyzed cohort.

The study cohort included adult kidney allograft recipients that participated in a study comparing the agreement between urine collection and albumin creatinine ratio for evaluation of urinary albumin excretion, termed UAE (11). Sixty-six patients were not included in the original report because their samples were intended to investigate albuminuria and obesity in transplanted patients, a study that was not completed. Our cohort included these patients, and the original cohort of 264 patients to create a cohort of 330 patients. Of these patients 16 had urinary albumin excretion of more than 300 mg per 24 h, 12 did not perform urine collection, three had no reliable data regarding collection timing or had inadequate collection and 13 had essential data missing. As a result, the final cohort of our study included 286 patients (Figure 1). Rabin medical center ethics committee approved the study. Informed consent was obtained from all participants and the study was conducted according to the declarations of Helsinki and Istanbul.

### Study Procedure

Urine collection was evaluated once for each patient. Patients were asked to provide a 24 h urine collection and were instructed to accurately monitor the collection time. When the duration of the collection differed from 24 h, the urine albumin was divided by the collection exact duration and multiplied by 24 so urine albumin excretion ration (UAER) was expressed per 24 h. Exclusion criteria were an infectious disease, use of non-steroidal anti-inflammatory drugs or acute rejection during the 30 days before the collection. Blood pressure (BP) was measured on the morning following the urine collection; BP is the mean of 3 measurements, taken by a trained personal following 5 min of complete rest, using an electronic oscillometric device. Gender, age, body weight and height were also recorded. Blood was drawn from a peripheral vein for serum creatinine measurement. The urinary albumin concentration was determined by a solid-phase, competitive chemiluminesence enzyme immunoassay (Immulite Analyzer, DPC). We revisited this cohort, 5 years after it was created, to evaluate time dependent CVD risk in relation to microalbuminuria.

### Outcomes

The primary outcome was the composite of MACE and all-cause mortality. The secondary outcomes included death censored MACE and the separate CV outcomes; coronary artery disease (CAD), cerebrovascular disease, peripheral vascular disease (PVD) and CV mortality. All-cause mortality was specified as primary outcome since the exact cause of death is sometimes complex, and the contribution of CV disease might be significant even when the cause of death is attributed to other factors. In addition, including all-cause mortality in the composite outcome reduced the risk of bias by competing risks.

CAD was defined as myocardial infarction, acute coronary syndrome and coronary artery revascularization percutaneously or by surgery. Cerebrovascular disease included cerebrovascular accident (CVA), transient ischemic attack (TIA), or carotid or cerebral revascularization, documented by emergency department referral or imaging. Peripheral vascular disease was defined as hospitalization due to peripheral ischemia, need for revascularization or need for amputation due to limb ischemia. Congestive heart failure (CHF) was defined according to echocardiogram evaluating new onset systolic heart function (or hospitalization attributed to CHF). Cardiovascular mortality was defined as mortality attributed to CVA, PVD, CAD, arrhythmia, or CHF.

All outcomes were collected from the patients' electronic health records (EHR) that included complete reports of all hospitalizations during the study period. Study researcher (DB) evaluated all reports for outcome events, in case of any doubt another researcher (BRZ) evaluated the report and in case of disagreement third researcher (RR) decided between the different opinions.

### Statistical Analysis

Data were analyzed using SPSS (version 25). For normally distributed variables results are expressed as mean and standard deviation (SD), and differences between the means of different groups were analyzed using standard *t*-test. To describe non-normally distributed variables we used median and interquartile range, and Mann-Whitney to show differences between groups. Comparison of frequency distribution was estimated by the χ2 test.

We used propensity stratified (PS) analysis to overcome confounding caused by differences in baseline characteristics. Propensity score was used by performing a forward stepwise logistic regression model with microalbuminuria as outcome. Variables considered in the model were gender, systolic blood pressure, donor type (living or deceased), immunosuppressive drug, LDL cholesterol, blood creatinine, anti-hypertensive medications [calcium channel blockers (CCB), beta blockers (BB), and angiotensin converting enzyme inhibitors (ACE I) or angiotensin receptor blockers (ARB)] and creatinine clearance.

For survival analysis we used Kaplan-Meier survival curve with log rank test for significance level. For adjusted analysis we stratified the analysis according to PS quartile.

We also used univariable and multivariable Cox proportional hazard model. The proportionality of hazard assumption was evaluated by examining the interaction between each variable and time. Multivariable analysis included traditional risk factors for CVD and albuminuria: age, gender, systolic blood pressure, LDL cholesterol, HbA1C, BMI, ACE I, or ARB use, blood creatinine and donor type.

The proportional hazard assumption for albuminuria was rejected. As a result, we used time varying Cox analysis investigating the first 2 years and the rest of the follow up time separately. This time point was chosen because the Kaplan Meier curve showed different hazard ratios before and after 2 years following the urine collection. The proportional hazard assumption for albuminuria was valid when evaluated for each time period separately.

For evaluation of subgroup interaction with the association between the exposure and the outcome, we used Cox analysis with microalbuminuria as time varying variable, and the interaction term between the subgroup and microalbuminuria and evaluated for significance of the interaction term.

## Results

Between December 2007 and February 2012, 330 urine collections were performed of them 286 patients were included in the analysis, their characteristics are presented in Table 1.

**Table 1:** Demographic, clinical, transplantation related, and laboratory characteristics of kidney transplanted patients who completed a timed urine collection.

	**All (286)**	**MIA (121)**	**No MIA (165)**	***p*-value**
Men *n* (%)	191 (67%)	83 (68%)	108 (65%)	0.613
Age (years)	53.0 ± 12.7	53.6 ± 12.6	52.6 ± 12.8	0.515
Diabetes *n* (%)	98 (34.3%)	42 (34.7%)	56 (33.9%)	0.9
HD *n* (%)	78 (27.3%)	36 (29.8%)	42 (25.5%)	0.424
CVA *n* (%)	33 (11.5%)	11 (9.1%)	22 (13.3%)	0.349
Living donor *n* (%)	192 (67.1%)	72 (59.5%)	120 (72.9%)	0.019
Primary renal disease				0.895
Glomerular	84 (29%)	32 (26.4%)	52 (31.5%)	
Diabetic nephropathy	39 (13.6%)	18 (14.9%)	21 (12.7%)	
Tubulointerstitial	34 (11.9%)	14 (11.6%)	20 (12.1%)	
Pkd	45 (15.7%)	19 (15.7%)	26 (15.8%)	
Nephrosclerosis	15 (5.2%)	8 (6.6%)	7 (4.2%)	
Unknown	69 (24.1%)	30 (24.8%)	39 (23.6%)	
Smoking *n* (%)	25 (8%)	9 (7.4%)	14 (8.5%)	0.828
Time from Tx (years)	1.2 (0.4–5.2)	1.5 (0.3–5.7)	1.2 (0.4–4.2)	0.253
Patients evaluated 1-year	158 (55%)	69 (57%)	89 (53.9%)	0.632
post transplantation				
**Medication**				
CCB *n* (%)	69 (24.1%)	38 (34.1%)	31 (18.8%)	0.017
Beta blockers *n* (%)	121 (42.3%)	61 (48.4)	60 (37.5%)	0.064
Duretics *n* (%)	37 (12.9&)	18 (14.9%)	19 (11.5%)	0.4
ACEI/ARB *n* (%)	104 (36.4%)	54 (44.6%)	50 (30.3%)	0.018
Tacrolimus *n* (%)	243 (85%)	97 (80%)	146 (88.5%)	
Cyclosphorine *n* (%)	14 (4.9%)	6 (5%)	8 (4.8%)	
MTOR inhibitors *n* (%)	29 (10.1%)	18 (15%)	11 (6.7)	0.071
**Indexes**				
Waist circumference (cm)	97.5 ± 16.6	99.5 ± 16.9	96 ± 16.2	0.087
BMI (kg/m^2^)	27.1 ± 5.1	27.9 ± 5.5	26.4 ± 4.7	0.014
Systolic BP (mmHG)	134.9 ± 18	140.3 ± 18.3	130.9 ± 16.9	<0.001
Diastolic BP (mmHG)	77.3 ± 12.7	78.1 ± 14.3	76.7 ± 11.4	0.367
**Laboratory**				
LDL (mg/dl)	89.2 ± 26.1	93.2 ± 27.3	86.3 ± 24.8	0.031
HDL (mg/dl)	52.3 ± 16.2	52.5 ± 15.2	52.1 ± 16.9	0.84
Creatinine clearance	76.3 ± 27.1	75.4 ± 30.9	77 ± 24.1	0.63
(ml/min)				
HbA1C (mg/dl)	6.1 ± 1	6.2 ± 1.1	6.1 ± 0.9	0.21

*Patients were grouped according to the presence of microalbuminuria. Percent relate to column, p-value describes the significance of statistical difference between groups according to a certain variable. Tx, transplantation*.

The mean 24 h urinary albumin excretion was 48.9 ± 63.3 mg [median 23 mg interquartile range (IQR) 9–56 mg]. Microalbuminuria was present in 121 patients (42.5%) and was associated with higher systolic blood pressure, use of antihypertensive medications, hyperlipidemia, higher blood creatinine, higher BMI, a living donor, and the use of mTOR inhibitors. By multivariable analysis all these factors, in addition to gender, were associated with microalbuminuria and used to calculate the propensity score.

Prevalence of primary renal disease was evaluated in both groups. However, since our institution serves as a tertiary referral center we rely on primary care physician investigation and diagnosis. We assume that glomerular disease, that tend to recur in the transplant, are usually diagnosed by biopsy. Other diseases such as PKD or diabetic nephropathy do not mandate invasive procedures unless diagnosis is unclear. There was no difference in the distribution of the different etiologies for renal failure between the groups (Table 1). As a result, we did not include this variable in the regression model.

### Association of MIA and Primary Outcome: CV Including All-Cause Mortality

Six patients (2.1%), of the 286 patients included in the cohort, were lost to follow up and hence excluded from the survival analysis.

We calculated the propensity score for microalbuminuria using the variables described above. The area under the receiver operating characteristic (ROC) curve for this model was 0.79 (0.73–0.84). The ROC curve analysis and Box plot of the four quartiles according to microalbuminuria status are presented in Supplementary Figures 1, 2, respectively.

**Figure 2:**
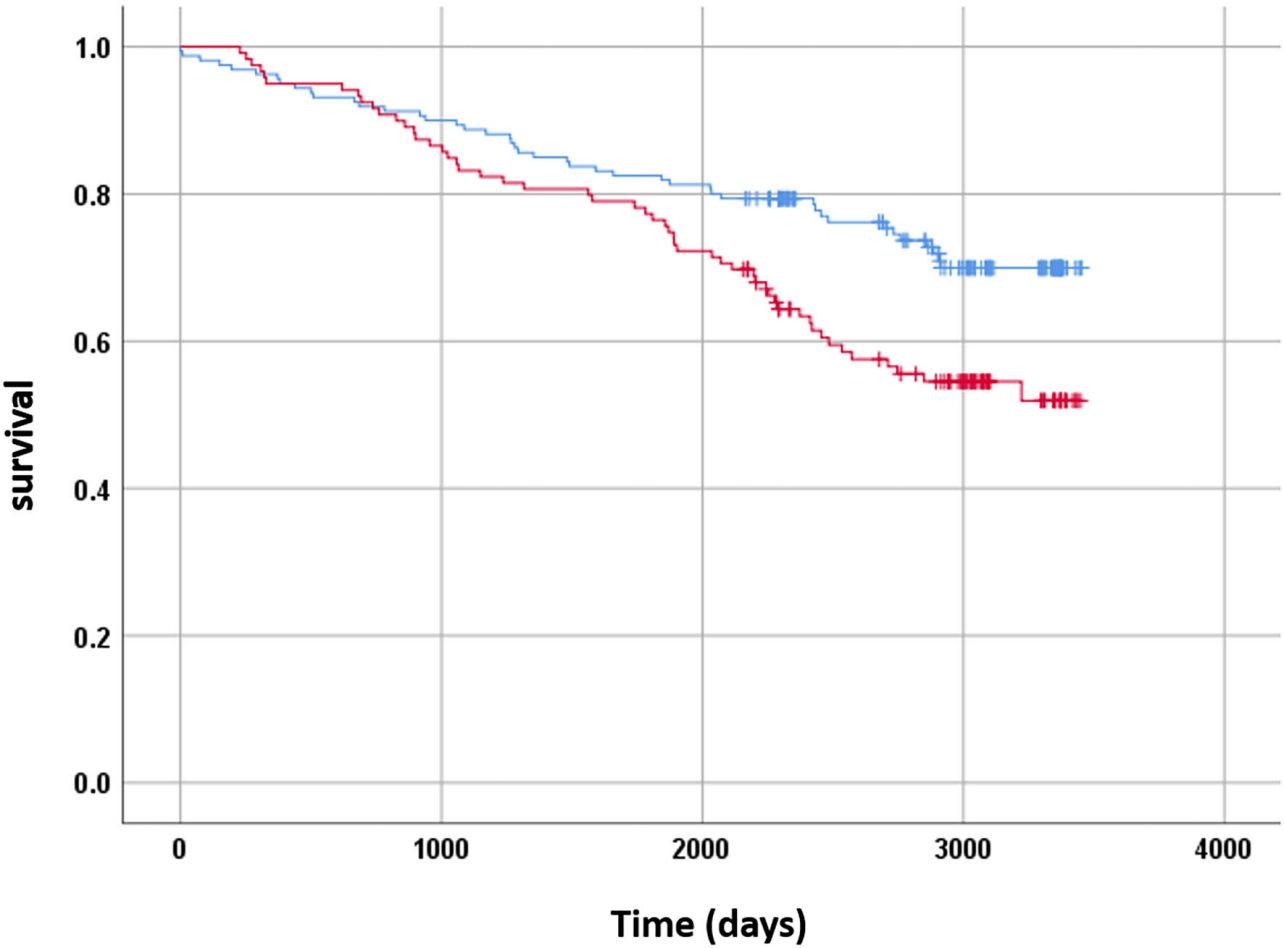
Kaplan-Meier analysis showing rates of CV outcome or death in relation to microalbuminuria. X axis shows days since urine collection, Y axis shows cumulative survival. Blue curve—patients without microalbuminuria, red curve—patients with microalbuminuria.

During median follow up time of 8.3 years (IQR 6.4–9.1) 144 outcome events occurred in 101 patients. The outcome events are described in the next section. By Kaplan-Meier analysis microalbuminuria was associated with increased rate of CV outcome or death (*p* = 0.03, Figure 2), the association was still significant after stratification according to PS quartiles (*p* = 0.048). By univariable time varying Cox proportional hazard analysis there was no association between microalbuminuria and CV outcomes during the first 2 years [Hazard Ratio (HR) 1.03, 95% Confidence Interval (CI) 0.43–2.43] but significant association appeared thereafter (HR 2.01, 95% CI 1.34–3.29). The association between microalbuminuria and CV outcomes after 2 years was significant even after stratification according to PS quartiles (HR 1.77, 95% CI 1.14–2.93). These results were not influenced by multivariable analysis for age, gender, systolic blood pressure, LDL cholesterol, HbA1C, BMI, blood creatinine, ACE I or ARB use and donor type (HR 1.76, 95% CI 1.05–2.96), which are traditional risk factors for CVD and albuminuria.

**Figure 3:**
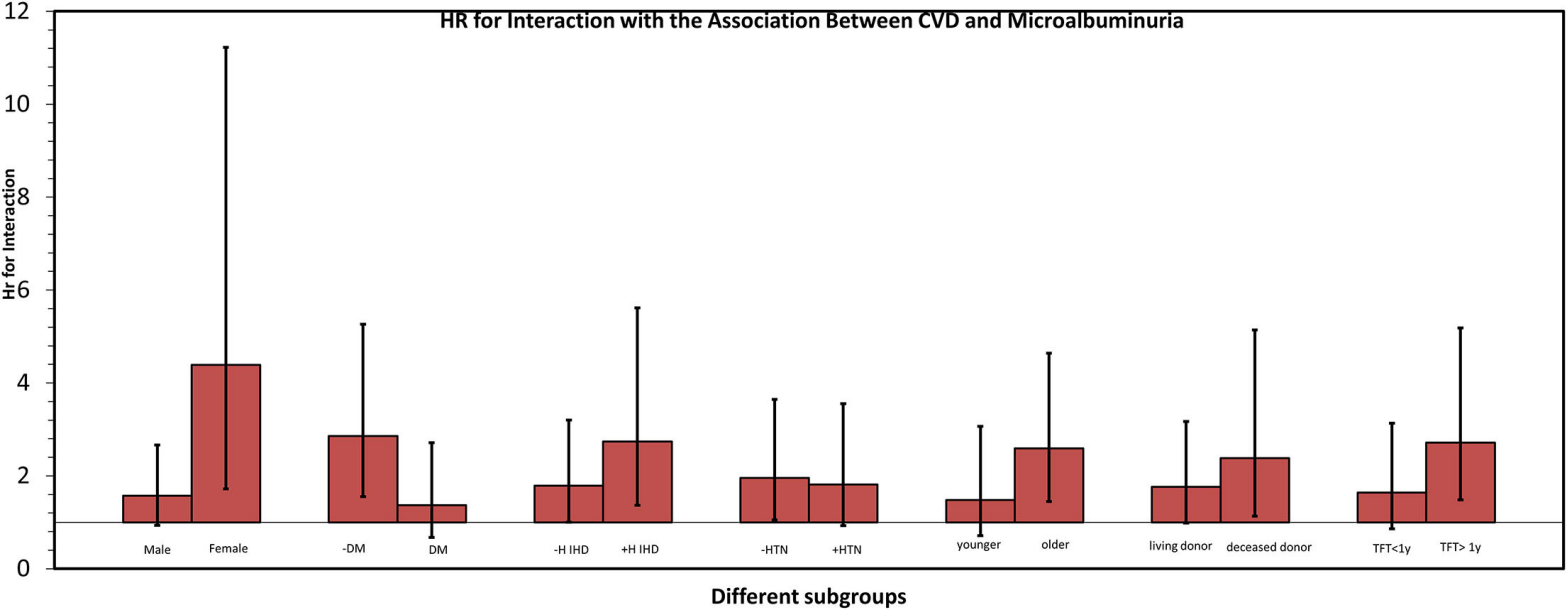
Subgroup analysis of CVD risk according to presence of microalbuminuria. Each couple presents a certain subpopulation according to a certain risk factor. Each column illustrates the relation between Hazard Ratio (HR) (Y axis) and presence of a risk factor (X axis). Black line across each column represents Confidence Interval for HR. None of the interactions were significant with *p* = 0.078, *p* = 0.295, *p* = 0.376, *p* = 0.803, *p* = 0.451, *p* = 0.366, and 0.245 for interaction with gender, Diabetes Mellitus (DM), history of heart disease, hypertension, time from transplantation, living donor, respectively, and median age (56 years). DM, Diabetes Mellitus; TFT, time from transplantation, >/< 1 y, more or less than a year; H IHD, history of ischemic heart disease. Older/younger—above and below median age (56.6 years).

Subgroup analysis for the main outcome of CV events and mortality is presented in Figure 3 and Supplementary Figure 1. There were some subgroups such as women, non-diabetics and patients with history of heart disease that exhibited an effect of microalbuminuria on survival, however no subgroup had significant interaction with the association between the main outcome and microalbuminuria (*p* = 0.078, *p* = 0.295, *p* = 0.376, *p* = 0.803, *p* = 0.451, *p* = 0.366 and 0.245 for interaction with gender, Diabetes Mellitus (DM), history of heart disease, hypertension, time from transplantation, living donor, and median age (56 years), respectively.

### Association of Microalbuminuria and Secondary Outcome Without All-Cause Mortality

Microalbuminuria was associated with increased risk for CV outcomes, without mortality, according to Kaplan-Meier curve (*p* = 0.008, Supplementary Figure 3), an association that was still significant after stratification according to PS quartiles (*p* = 0.045). This association became significant according to time varying analysis after 2 years from documentation of albuminuria, by simple and PS stratified analyses [(HR 2.05, 95% CI 1.26–3.33) and (HR 1.79, 95% CI 1.05–3.08)], respectively. It was still significant using multivariable analysis adjusted for widely accepted CV risk factors including gender, systolic blood pressure, LDL cholesterol, HbA1C, blood creatinine and donor type (HR 1.74, 95% CI 1.04–2.91).

### Association of Microalbuminuria and Secondary Outcomes: CAD, CVA, PVD, and All-Cause Mortality

Supplementary Table 1 depicts the different components of secondary outcome in relation to microalbuminuria. During follow up there were 53 episodes of CAD, 24 (14.9%) in the patients without microalbuminuria and 29 (24.3%) in the microalbuminuria group. Microalbuminuria was associated with CAD 2 years after timed urine collection by simple and PS stratified analyses (HR 2.36, 95% CI 1.29–4.342) and (HR 1.79, 95% CI 1.05–3.08), respectively, according to time dependent Cox analysis. During the first 2 years of follow up there was no increased risk of CAD.

There were 25 episodes of CVA, 10 (8.3%) in the microalbuminuria group and 15 (9.1%) in the non-microalbuminuria group. By Cox model there was no difference in the hazard for CVA between the groups (HR 1, 95% CI 0.55–2.25).

In contrast, PVD occurred in 12 (9.9%) patients in the microalbuminuria group and 4 (2.4%) in the non-microalbuminuria group. Simple cox model revealed a significantly increased risk for PVD associated with microalbuminuria (HR 4.17, 95% CI 1.34–12.93). However, after stratification according to PS quartiles the association was no longer significant (HR 3.15, 95% CI 0.95–10.58).

During follow up there were 42 events of all-cause mortality, 22 of them (18.6%) in the microalbuminuria group and 20 (12.4%) in the control group. There was no statistically significant association between microalbuminuria and mortality (HR 1.44, 95% CI 0.79–2.65).

## Discussion

We evaluated the association between microalbuminuria, as measured by accurate urine collection, and the composite outcome of CVD and all-cause mortality, following kidney transplantation. We found time varying association between microalbuminuria and CVD morbidity and mortality, with increased risk emerging around 2 years post documentation of albuminuria. The results were similar for the secondary outcome of CVD excluding non-cardiovascular death. The increased risk of CV events in the presence of albuminuria was driven mainly by CAD and PVD cases, while CVA incidence was similar between groups.

Our results are in accordance with previous studies in various populations that showed an association between microalbuminuria and CVD. The first hint to this phenomena in kidney transplanted patients was observed was in a paper describing microalbuminuria as a cause of graft loss and death (8). A few years later, Weiner and colleagues were the first to design a study dedicated to this hypothesis. They evaluated the association between CVD and various degrees of albuminuria as assessed by albumin-creatinine ratio. Even though they did not reach statistical significance, probably due to short follow-up, they raised awareness to the rising risk in this special population (9). A similar project published this year form our institute exemplifies the importance of controlling proteinuria to reduce CVD risk (10).

The attenuated effect described by Weiner et al. might result from the use of ACR, which could be confounded by creatinine excretion, a measure affected by gender, BMI and age. This study also assessed incidence of CVD close to specimen collection, hence did not have the perspective of long-term follow-up.

Attention should be given to other contributing factors and possible confounders that arise during analysis. In a recent paper by Rahamimov et al. BMI was associated with microalbuminuria by univariate logistic regression analysis, yet after multivariate analysis this relation lost significance. BMI was also associated with increased odds ratio [OR] for microalbuminuria (12), as well as serum creatinine and treatment with mTOR inhibitors.

Multivariate logistic regression analysis, in our cohort, showed that treatment with mTOR inhibitor, systolic blood pressure, serum creatinine, and BMI were associated with increased OR for microalbuminuria. BMI was still significantly associated with increased rate of urinary albumin excretion after introducing DM status into the model. The influence of adiposity on albuminuria, if it occurs, is expected to be time-related and to develop after prolonged exposure to an obese environment provided by the recipient. Risk of albuminuria was shown to correlate with high BMI 3 years following transplantation (12), however this influence was not evident in our cohort because of the shorter time line between transplantation and urine collection. When BMI was included in the multivariate analysis it did not affect the association between microalbuminuria and CVD.

Several mechanistic explanations were suggested to elucidate in the late period, BMI is an independent predictor of albuminuria and microalbuminuria after adjustment, amongst others, for arterial pressure and the presence of DM. The association between microalbuminuria and cardiovascular risk. One of them poses albuminuria as a marker of diffuse atherosclerosis, a major cause for CVD in transplanted patients (13–15). As atherosclerosis develops over years and most patients included in the study had relatively short follow up, this explanation falls in short. Another optional mechanism is that albuminuria activates neuro-hormonal response in the kidney that may increase the CV risk (16). This mechanism is possible as the timeline for nerve regeneration in transplanted kidneys that causes hypertension is 2 years (17), a similar period was observed by us as the lag between albuminuria and CVD morbidity. Nerve density reaches values observed in native kidney arteries after 2 years and is associated with hypertension-related arteriolar lesions in transplanted kidneys (17).

One possible contributory factor could be glomerular hyperfiltration. The solitary functioning kidney carries the metabolic burden alone, and more so in the presence of increased weight and BMI as seen in the MIA group. Among 9,515 healthy participants from the CARTaGENE trial (18), 473 had glomerular hyperfiltration. Compared with the normal filtration rate, glomerular hyperfiltration was associated with an increased cardiovascular risk (HR 1.88). The cardiovascular risk of the highest eGFR percentiles was similar to that of participants with CKD3a. These findings suggest that glomerular hyperfiltration is independently associated with increased cardiovascular risk in middle-aged healthy individuals. Yet, it is hard to evaluate hyperfiltration in a transplanted kidney as the baseline potential of the transplanted kidney is variable, and this hypothesis should be addressed in future studies. Yet, there was a difference in the donor type between groups, with a deceased donor more prevalent among patients with microalbuminuria. This difference might be due to mismatch between the transplanted kidney and the recipient metabolic demand. However, this explanation is speculative and should be address in future studies.

One reasonable pathway linking albuminuria with CVD could be endothelial dysfunction, that might link both phenomena. Endothelial dysfunction was characterized in diabetic patients, as well as the general population (19), by various tests including endothelial function plasma markers (20) and vasodilation in response to agonists such as cholinergic factors and increase in flow (21). However, as our study didn't evaluate any measure of endothelial dysfunction, this is a purly hypothetical association and further research is required to evaluate the role of endothelial dysfunction after kidney transplantation.

The reason for the time varying nature of this association is not clear and further studies are needed to answer this question. A possible explanation is that there is a different threshold of damage accumulation, needed for each manifestation. Common biological pathways can cause both microalbuminuria and CVD but at different time course, i.e., rapid development of microalbuminuria and slower for CVD. Since urine collection was performed chronologically soon after transplantation (median time of 2.8 years) this could be a reasonable interpretation. Longitudinal studies evaluating change in urine albumin excretion over time, and its association with cardiovascular risk, might shed some light on this important observation.

Our study has several important strengths. First, for albuminuria evaluation, we used very accurate timed urine collection, in a research setting and under strict observation. This prevented the confounding effects of both adjustment to creatinine as mentioned above and imprecise collection that is very common in the clinical setting. Another advantage we employed was the use of EHR as a reliable information source to report cardiovascular events, and their evaluation by experienced physicians. The use of EHR also enabled detailed analysis of the different CV outcomes. Last, our cohort was followed for an extensive time period, enabling the discussion of causality between a known risk factor and disease progression. These characteristics are unique and provide both insight and strength to our findings, and reduce bias resulting from incomplete reporting.

This study has also some limitations. First, our findings suggest different association for the various components of the secondary outcome. Unlike CAD and PVD that were associated with microalbuminuria this was not the case for CVA. Composite CV outcome are a common and powerful tool, but there is some difference in the pathophysiology of the different components and detailed analysis might be of value. These differences should be evaluated in larger studies and in other populations, in search for mechanistic distinction.

Second, our small size population attenuates the study power. As accurate urine collection is cumbersome and time consuming, it is hard to use in large sample groups. Due to that, subgroup analysis was limited and valuable data regarding interactions between microalbuminuria and CVD could not be interpreted. Another limitation is that this is a single center study which might limit its external validity especially regarding non-Caucasians populations that were not represented in the analysis.

Changes in GFR over time with progressive CKD, could account for the increase rate of MACEs rather than microalbuminuria. Unfortunately, serial GFR evaluations were no available for our cohort. However, a recently published evaluation of the effect of time dependent proteinuria on CVD found no independent association between serial GFR measurements and MACE (10).

Of note, prevalence of primary renal disease was similar between groups, yet as a tertiary referral center, we rely on primary care physician evaluation and diagnosis and primary renal disease prevalence should be interpreted cautiously.

Last, the interaction of CVD with time was not pre specified and was discovered when the proportionality assumption was evaluated before performing Cox analysis. Thus, this observation should be interpreted cautiously until verified by other studies. Nevertheless, the time varying nature might give important insights regarding the association of microalbuminuria and CVD, as a continuum of the same pathological process.

According to our results, and studies evaluating other populations, microalbuminuria is an independent risk factor for CVD, and a marker of it (22–24). Chronic kidney disease is a risk factor for CVD (25–27) and following kidney transplantation it is added to the use of medication endangering with hypertension and diabetes, thus increasing the risk. Clinicians should perceive microalbuminuria as a risk equivalent when evaluating their patients' CVD risk and initiate primary or secondary preventive measures.

Further research is needed in order to extend our understanding of the association between albuminuria and CVD. These should rely on the association between urine albumin excretion using urine collection to verify our results. large scale studies are not expected due to the relative complexity of accurate timed urine collection, but even small series can be pooled together to generate statistical power. Another important aspect that should be explored is the dynamic of urine albumin excretion and its association with CVD. These kinds of studies are especially important due to the time varying nature of the association observed in our study. Furthermore, a mechanistic explanation should be sought to reveal the relation between albuminuria and different time points after kidney transplantation, by using urine exosome extraction technique. Since the origin of the endothall differ, from recipient vs. donor, this could be a non-invasive manor to assess pathology emergence.

## Conclusion

We show that microalbuminuria is associated with increased risk of CVD in kidney transplanted patients, 2 years after a timed urine collection documenting its appearance. Therefore, newly documented albuminuria after kidney transplantation should be considered risk equivalent for CVD.

## Data Availability Statement

The data analyzed in this study is subject to the following licenses/restrictions: deidentified database will be provided from the corresponding author upon a specified request. Requests to access these datasets should be directed to benayarz@gmail.com.

## Ethics Statement

The studies involving human participants were reviewed and approved by Rabin Medical Center ethics committee approved the study. Informed consent was obtained from all participants and the study was conducted according to the declarations of Helsinki and Istanbul. The patients/participants provided their written informed consent to participate in this study.

## Author Contributions

DB performed data collection, decision making regarding clinical outcome per patient, designed the figures, and wrote the manuscript with BR. RR, BZ, and LA-G performed research conduction and contributed to the final version of the manuscript. AC was involved in data collection and analysis of the baseline data. BR conceived the presented idea, performed the statistical analysis, designed the figures, and wrote the manuscript with DB. All authors discussed the results and contributed to the final manuscript.

## Conflict of Interest

BR received consultation fee from Fresenius medical care and AstraZenca. The remaining authors declare that the research was conducted in the absence of any commercial or financial relationships that could be construed as a potential conflict of interest.

## References

[1] Wang J Wang F Liu S Zhou M Zhang L Zhao M. Reduced kidney function, albuminuria, and risks for all-cause and cardiovascular mortality in china: a population-based cohort study. BMC Nephrol. (2017) 18:188. 10.1186/s12882-017-0603-9 28592243 PMC5463353

[2] Rebholz CM Coresh J Ballew SH McMahon B Whelton SP Selvin E. Kidney failure and ESRD in the atherosclerosis risk in communities (ARIC) study: comparing ascertainment of treated and untreated kidney failure in a cohort study. Am J Kidney Dis. (2015) 66:231–9. 10.1053/j.ajkd.2015.01.016 25773483 PMC4516566

[3] Joshi S Viljoen A. Renal biomarkers for the prediction of cardiovascular disease. Curr Opin Cardiol. (2015) 30:454–60. 10.1097/HCO.0000000000000177 26049396

[4] Russo LM Sandoval RM Campos SB Molitoris BA Comper WD Brown D. Impaired tubular uptake explains albuminuria in early diabetic nephropathy. J Am Soc Nephrol. (2009) 20:489–94. 10.1681/ASN.2008050503 19118149 PMC2653682

[5] Rossing P Persson F Frimodt-Møller M. Prognosis and treatment of diabetic nephropathy: recent advances and perspectives. Nephrol Ther. (2018) 14(Suppl. 1):S31–7. 10.1016/j.nephro.2018.02.007 29606261

[6] Konno S Munakata M. Moderately increased albuminuria is an independent risk factor of cardiovascular events in the general Japanese population under 75 years of age: the Watari study. PLoS ONE. (2015) 10:e0123893. 10.1371/journal.pone.0123893 25849735 PMC4388624

[7] Tanaka F Komi R Makita S Onoda T Tanno K Ohsawa M. Low-grade albuminuria and incidence of cardiovascular disease and all-cause mortality in nondiabetic and normotensive individuals. J Hypertens. (2016) 34:506–12. 10.1097/HJH.0000000000000809 26820477

[8] Halimi J-M Matthias B Al-Najjar A Laouad I Chatelet V Marlière J-F. Respective predictive role of urinary albumin excretion and nonalbumin proteinuria on graft loss and death in renal transplant recipients. Am J Transplant. (2007) 7:2775–81. 10.1111/j.1600-6143.2007.02010.x 17949457

[9] Weiner DE Park M Tighiouart H Joseph AA Carpenter MA Goyal N. Albuminuria and allograft failure, cardiovascular disease events, and all-cause death in stable kidney transplant recipients: a cohort analysis of the FAVORIT trial. Am J Kidney Dis. (2019) 73:51–61. 10.1053/j.ajkd.2018.05.015 30037726 PMC6309643

[10] Molcho M Rozen-Zvi B Shteinmats T Ben Dor N Vahav I Nesher E. Temporal changes of proteinuria after kidney transplantation: association with cardiovascular morbidity and mortality. J Nephrol. (2020) 33:1059–66. 10.1007/s40620-020-00703-6 31953621

[11] Erman A Rahamimov R Mashraki T Levy-Drummer RS Winkler J David I. The urine albumin-to-creatinine ratio: assessment of its performance in the renal transplant recipient population. Clin J Am Soc Nephrol. (2011) 6:892–7. 10.2215/CJN.05280610 21212424 PMC3069384

[12] Zingerman B Erman A Mashraki T Chagnac A Rozen-Zvi B Rahamimov R. Association of obesity and muscle mass with risk of albuminuria in renal transplant recipients. J Nephrol. (2020) 10.1007/s40620-020-00883-1. [Epub ahead of print]. 33098523

[13] Carpenter MA Weir MR Adey DB House AA Bostom AG Kusek JW. Inadequacy of cardiovascular risk factor management in chronic kidney transplantation - evidence from the FAVORIT study. Clin Transplant. (2012) 26:E438–46. 10.1111/j.1399-0012.2012.01676.x 22775763 PMC4388027

[14] Pilmore HL Skeans MA Snyder JJ Israni AK Kasiske BL. Cardiovascular disease medications after renal transplantation: results from the Patient Outcomes in Renal Transplantation study. Transplantation. (2011) 91:542–51. 10.1097/TP.0b013e31820437bd 21301401

[15] Park M Shlipak MG Vittinghoff E Katz R Siscovick D Sarnak M. Associations of kidney injury markers with subclinical cardiovascular disease: the Multi-Ethnic Study of Atherosclerosis. Clin Nephrol. (2015) 84:358–63. 10.5414/CN108668 26558369 PMC4776253

[16] Afonso L Zalawadiya SK Veeranna V Panaich SS Niraj A Jacob S. Relationship between red cell distribution width and microalbuminuria: a population-based study of multiethnic representative US adults. Nephron Clin Pract. (2011) 119:c277–82. 10.1159/000328918 21921640

[17] Mauriello A Rovella V Borri F Anemona L Giannini E Giacobbi E. Hypertension in kidney transplantation is associated with an early renal nerve sprouting. Nephrol Dial Transplant. (2017) 32:1053–60. 10.1093/ndt/gfx069 28498963 PMC5837349

[18] Dupuis M-E Nadeau-Fredette A-C Madore F Agharazii M Goupil R. Association of glomerular hyperfiltration and cardiovascular risk in middle-aged healthy individuals. JAMA Netw Open. (2020) 3:e202377. 10.1001/jamanetworkopen.2020.2377 32275320 PMC7148438

[19] Chen J Hamm LL Mohler ER Hudaihed A Arora R Chen C-S. Interrelationship of multiple endothelial dysfunction biomarkers with chronic kidney disease. PLoS ONE. (2015) 10:e0132047. 10.1371/journal.pone.0132047 26132137 PMC4488859

[20] Fu J Lee K Chuang PY Liu Z He JC. Glomerular endothelial cell injury and cross talk in diabetic kidney disease. Am J Physiol Renal Physiol. (2015) 308:F287–97. 10.1152/ajprenal.00533.2014 25411387 PMC4329492

[21] Cosson E Pham I Valensi P Pariès J Attali J-R Nitenberg A. Impaired coronary endothelium-dependent vasodilation is associated with microalbuminuria in patients with type 2 diabetes and angiographically normal coronary arteries. Diabetes Care. (2006) 29:107–12. 10.2337/diacare.29.01.06.dc05-1422 16373905

[22] Xia F Liu G Shi Y Zhang Y. Impact of microalbuminuria on incident coronary heart disease, cardiovascular and all-cause mortality: a meta-analysis of prospective studies. Int J Clin Exp Med. (2015) 8:1–9. 25784968 PMC4358423

[23] Scheven L Halbesma N de Jong PE de Zeeuw D Bakker SJL Gansevoort RT. Predictors of progression in albuminuria in the general population: results from the PREVEND cohort. PLoS ONE. (2013) 8:e61119. 10.1371/journal.pone.0061119 23723966 PMC3664562

[24] Fung CSC Wan EYF Chan AKC Lam CLK. Association of estimated glomerular filtration rate and urine albumin-to-creatinine ratio with incidence of cardiovascular diseases and mortality in Chinese patients with type 2 diabetes mellitus - a population-based retrospective cohort study. BMC Nephrol. (2017) 18:47. 10.1186/s12882-017-0468-y 28152985 PMC5290675

[25] Hart A Weir MR Kasiske BL. Cardiovascular risk assessment in kidney transplantation. Kidney Int. (2015) 87:527–534. 10.1038/ki.2014.335 25296093

[26] Kolonko A Chudek J Szotowska M Kuczera P Wiecek A. Cardiovascular risk factors and markers of atherosclerosis in stable kidney transplant recipients. Transplant Proc. (2016) 48:1543–50. 10.1016/j.transproceed.2015.12.134 27496444

[27] Rao NN Coates PT. Cardiovascular disease after kidney transplant. Semin Nephrol. (2018) 38:291–7. 10.1016/j.semnephrol.2018.02.008 29753404

