# Supplementary material for: Microalbuminuria After Kidney Transplantation Predicts Cardiovascular Morbidity
Source: Front Med (Lausanne). 2021 Apr 12;8:635847. doi: 10.3389/fmed.2021.635847 (PMC8071984; doi:10.3389/fmed.2021.635847)

Supplamentary Data

Supplementary table 1 – Outcome events. Elaborated documentation of events from HER in the two different groups of transplanted patients, divided according to presence of MIA. CAD – Coronary Artery Disease, CVA – Cerebro-Vascular Accident, PVD – Peripheral Vascular Disease. HR – hazard Ratio. Percent relate to columns population.

| HR | NO MIA (165) | MIA (121) |  |
| --- | --- | --- | --- |
| 2.36 | 25 (14.9%) | 29 (24%) | CAD |
| 1 | 15 (9.1%) | 10 (8.3%) | CVA |
| 4.17 | 4 (2.4%) | 12 (9.9%) | PVD |
| 1.46 | 20 (12.4%) | 23(18.6%) | All Cause Mortality |

Supplementary figure S1 – ROC curve describing the logistic regression model for the propensity score with MIA as an outcome (blue curve).


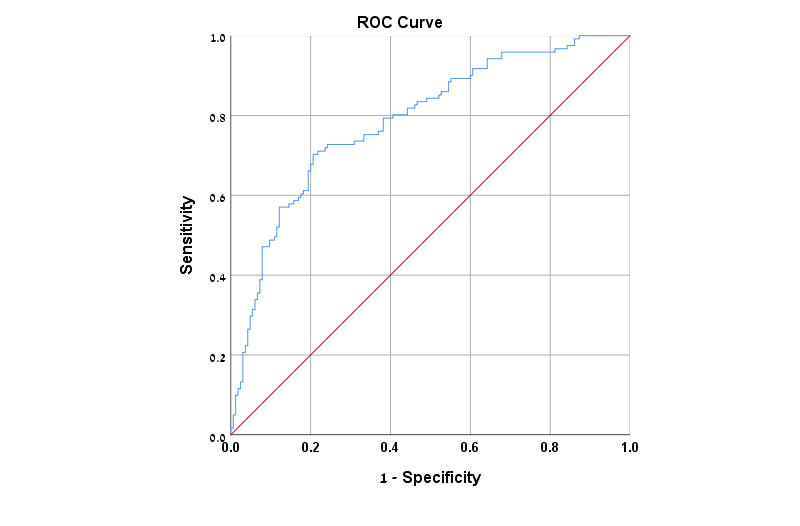


Supplementary figure S2 – propensity score distribution according to quartiles. The bars show the median and inter-quartile range. Blue bars – patients without MIA, red bars – patients with MIA
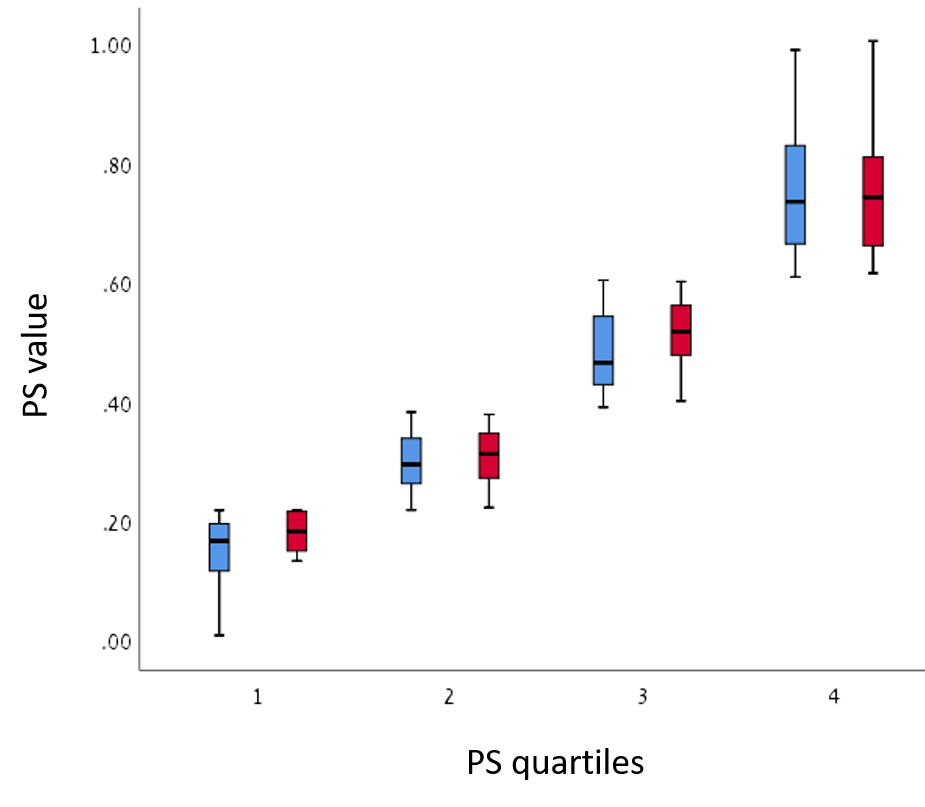


Supplementary figure S3: Kaplan-Meier curve showing association of MIA with increased risk of CV outcomes (p=0.005) without mortality. Blue curve – patients without MIA, red curve – patients with MIA. P represents p-value of significant difference between the two slops.


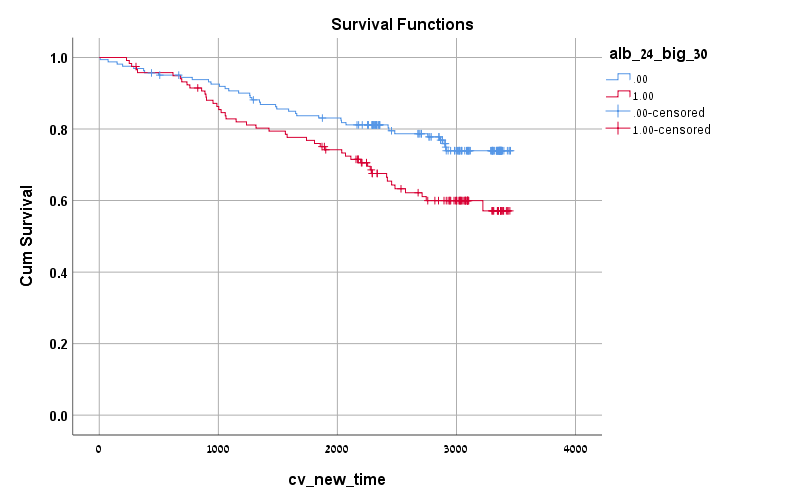

Supplement: Supplementary file 1 [file Data_Sheet_1.docx]
